# Fetal macrosomia and its associated factors among pregnant women delivered at national referral hospital in Uganda, a case-control study

**DOI:** 10.1371/journal.pone.0294543

**Published:** 2025-04-21

**Authors:** Peter Wanyera, Eve Nakabembe, Mike Nantamu Kagawa

**Affiliations:** 1 Department of obstetrics and gynaecology, School of Medicine, College of Health Sciences, Makerere University, Kampala, Uganda; 2 Busiu Health Centre IV, Mbale District Local Government, Mbale, Uganda; University of Melbourne, AUSTRALIA

## Abstract

**Background:**

The delivery of macrosomic newborns (newborns>4000gm) is increasing steadily worldwide and yet it is associated with many complications. The risk factors for fetal macrosomia include weight at first antenatal visit, previous delivery of a large newborn, newly diagnosed diabetes in pregnancy, increasing number of deliveries, a male fetus, and many others. There is paucity of data on fetal macrosomia in Uganda and the objective of this study therefore was to determine the risk factors for fetal macrosomia among women who delivered at a National Referral Hospital in Kampala, Uganda..

**Methods:**

An unmatched case-control study was conducted among 177 cases and 354 controls at Kawempe National Referral Hospital. Data was collected using interviewer-administered questionnaires for three months from 8^th^ February 2021–25^th^ May 2021. Comparison between the cases and the controls was done using the student t-test for normally distributed continuous data. The Mann Whitney U Test (Wilcoxon Rank Sum Test) was used to compare data that was not normally distributed. Binary logistic regression was used in bivariate and multivariate analysis for factors associated with fetal macrosomia using STATA version 16.0.

**Results:**

A total of 531 participants were recruited into the study in a ratio of one case to two controls. The mean age for cases was 28.5 ± 5.9 years and 25.9 ± 5.4 years for controls while the mean weight was 78.4 ± 12.4Kgs for cases and 68.2 ± 11.7 Kg for controls. Risk factors for fetal macrosomia included maternal age ≥40 years (aOR = 7.4, [95%CI 1.37–39.44], p value = 0.020), maternal weight ≥80 kg (aOR = 4.0, [95%CI 2.15–7.40], p value <0.001), maternal height ≥160 cm (aOR = 1.6, [95%CI 1.02–2.51], p value = 0.040), being married (aOR = 2.55, [95%CI 1.08–6.06], P value = 0.038), gestation age ≥40 weeks (aOR = 1.8,[95%CI 1.16–2.82], p value = 0.009), previous macrosomia (aOR = 2.2, [95%CI 1.26–3.81], p value = 0.006) and male babies (aOR = 1.78, [95%CI 1.14–2.77], p value = 0.011).

**Conclusions:**

The main risk factors for fetal macrosomia were higher maternal weight and height, advanced age as well as post-datism, previous delivery of a large newborn, male fetus and being in a marital relationship. A well-designed protocol to identify women with risk factors for fetal macrosomia may help to provided targeted interventions in this group.

## Introduction

Globally, macrosomia affects 3–15% of all pregnancies [1] and in high income countries, the magnitude of macrosomia ranges from 5 to 20% of all births [2]. The worldwide prevalence of birth of infants greater or equal to 4000gm is approximately 9 percent and 0.1 percent for weight greater or equal to 5000g, with wide variations among countries [3]. In the United States, approximately 7 percent of live born infants weigh greater than or equal to 4000 g and 1 percent weigh greater than 4500g [4]. The prevalence of birth weight greater than or equal to 4000 g in low-income countries is typically 1–5 percent but ranges from 0.5 to 14.9 percent [5]. Recent evidence suggests that the incidence of macrosomia is increasing. For example, findings from a study done in Ethiopia in 2014 showed a prevalence of macrosomia of 6.7% [6]. This is attributed to the increase in maternal anthropometry due to overnutrition, reduced cigarette smoking, and changes in socio -demographic factors such as urbanization [7,8]. Management of fetal macrosomia has for long been an obstetric challenge and is becoming an increasingly important problem because of the rising incidence of macrosomia and the associated risks to the mother and infant.

Documented risk factors for macrosomia in developing countries can be attributed to the rapid nutrition transition leading to increased weight at first visit (maternal obesity), excessive weight gain in pregnancy, and gestational diabetes [8,9]. Other factors associated with fetal macrosomia include genetics, race, ethnicity, multiparity, advanced maternal age, duration of gestation (post-datism), previous macrosomic infant, male fetus and diabetes mellitus types I and II [10].

Fetal macrosomia complicates the delivery process for both mothers and neonates and macrosomic babies have higher rates of developing both short- and long-term adverse health outcomes [1]. Fetal and neonatal outcomes of fetal macrosomia include shoulder dystocia, birth trauma like brachial plexus injury and skeletal injury, chorioamnionitis because of prolonged labour, meconium aspiration, low APGAR score, neonatal hypoglycemia and intrauterine fetal death [8,11]. Most studies done on fetal macrosomia in Africa were done in Ethiopia and there is paucity of information on this subject in Uganda.

Since prior diagnosis/ antenatal prediction of fetal macrosomia is challenging both by sonography and usual estimation by palpation, paying attention to detail in terms of risk assessment based on factors such as maternal weight, height, previous history in terms of birth outcome and birth weights as well as tests like blood sugar levels, could help identify pregnant women at increased risk of macrosomia. [12]. The aim of this study was to determine the risk factors for fetal macrosomia among women who delivered at Kawempe National Referral Hospital (KNRH) in Kampala, Uganda. It is hoped that with this information, it will be possible to suggest ways in which prediction of macrosomia can be improved in order to institute timely interventions to minimise or mitigate the adverse maternal and fetal complications associated with macrosomia.

## Materials and methods

This was an unmatched case-control study conducted among postpartum women on the labour and post-natal wards at KNRH in Kampala. KNRH is one of the largest and public national referral hospitals in Uganda. It is a teaching hospital for the department of Obstetrics and Gynecology of Makerere University College of Health Sciences. The hospital is located approximately 12 kilometers from Kampala capital city centre and has a bed capacity of 170. The population of Kampala City where the hospital is located is about 2 million people during the night, but this rises to about 4.5 million during the day. Among 60–80 daily deliveries at this facility, there may be 2–3 macrosomic infants. Most patients received at the hospital are from within the city and the surrounding districts with a few referrals from lower health units all over the country.

### Characteristics of participants

Participants were women who had delivered at KNRH during the study period. Women regardless of parity or age who had delivered babies of birth weight greater or equal to 4000gm were taken as cases and those with babies weighing 2500 kg – 3990 kg were considered as controls.

### Sample size determination

The sample size was estimated using an online formula for case control studies by Glaziou Phillipe [13].

This was based on a study done in Ethiopia by Wondie et al to determine factors associated with macrosomia among neonates delivered at Debre Markos Referral Hospital, Northwest Ethiopia in 2014 [14]. In this study, among a subgroup of multiparous women, 70.7% were cases and 60.1% were controls. Using the following assumptions: Odds ratio 1.9, Exposed controls 60.1%, Alpha risk 5%, and a Control/ Case ratio 2:1, the number of cases required was determined to be 177, and the number of controls 354; giving an overall sample size of 531 participants.

### Sampling method

The delivery register was used to find the names of mothers who had delivered babies of 4000mg or more and these were selected consecutively as cases. For each case, two controls were selected as the mothers who had delivered, during the same period, a baby weighing between 2500–3999gm, just before and after the case as indicated in the delivery register giving a ratio of cases: controls = 1:2. In the event that any of the selected controls as per the register was found to be a case, then four controls were selected, that is two before and two after the two cases.

During sampling, which was done consecutively from 8th February 2021–25th May 2021, mothers who were identified as cases or controls were approached and given a full explanation about the study. Following acceptance to participate in the study, written informed consent was obtained. For minors (emancipated or non-emancipated), consent was sought from a legal guardian or parent present at the time of recruitment. Data collection was done within 24hours of delivery using a pre-tested interviewer administered questionnaire. Additional Information was obtained from the mother’s clinical chart/delivery report where such information was documented.

Maternal weight was taken at the time of data collection (after delivery of the baby) while the mother was still on the ward. Gestation age was determined based on the mother’s last normal menstrual period found in the patient’s record and date of delivery. All these were collected as continuous variables and categorization was done at analysis. Deliveries that happened between 37–39 weeks were considered to be term while those from 40–42 weeks were considered as post-term. Parity was grouped into primiparous if the mother had the current delivery as the first one, multiparous if she had two to four deliveries or grand-multiparous if she had more than five deliveries including the current one.

### Data collection and analysis

Data was collected using pre-tested interviewer administered structured questionnaires for three months from 8^th^ February 2021–25^th^ May 2021 when the expected sample size was achieved. A database was designed using the computer software EPI-DATA version 4.6 for data. The raw data was securely stored to maintain confidentiality. The data was then exported to STATA version 16.0 software for analysis. For continuous data that was normally distributed, comparison between the cases and the controls was done using the student t-test. For data that was not normally distributed, comparison between the cases and the controls was done using the Mann Whitney U Test (Wilcoxon Rank Sum Test). Binary logistic regression was used in multivariate analysis for factors associated with fetal macrosomia. All variables with a P-value <0.05 using the chi-square test were included in the multivariable model. Backward stepwise selection (or backward elimination) method was used in analysis for the associated factors. Odds ratios were used as the measure of association and reported along with their 95% confidence intervals. Normality was tested by examining the descriptive statistics to see if the data exhibited minimal asymmetry compared to a normal distribution curve.

### Ethics approval and consent to participate

Approval was obtained from the Makerere University School of Medicine Research and Ethics Committee (#REC REF 2021–002). Voluntary written informed consent was sought from each participant before enrolment into the study. If the participant was a minor, the parent or legal guardian was requested to provide an informed consent in writing before the participant was enrolled into the study. The study participants were informed of their right to refuse participation or withdraw from the study at any time if they so wished, and that refusal to participate or withdrawal of consent from the study would not result in any penalties or have any impact on their care in the hospital.

## Results

A total of 531 participants were recruited into the study with 177 (33.3%) as cases and 354 (66.7%) as controls making up a ratio of 1:2. The mean MUAC and BMI was significantly higher among the cases than the controls. The maternal age, weight and height was significantly higher among the cases compared to the controls across all the categories (Table 1).

**Table 1:** Maternal characteristics.

	Cases n=177	Controls n=354	P value
Maternal age in years (mean ± SD) Maternal weight (kgs) (mean ± SD) Maternal height (cm) (mean ± SD) MUAC (cm) (mean ± SD) ^BMI (kg/m²) (mean ± SD)^	28.5 ± 5.9 78.4 ± 12.4 163.0 ± 6.9 30.6 ± 3.3 29.5 ± 4.5	25.9 ± 5.4 68.2 ± 11.7 159.5 ± 7.6 28.3 ± 3.4 26.8 ± 4.2	<0.001 <0.001
**Maternal age, year categories**			**<0.001**
<30	109 (61.6)	264 (74.6)	
30–39	59 (33.3)	88 (24.9)	
≥40	9 (5.1)	2 (0.5)	
**Weight categories (kg)**			**<0.001**
<80	98 (55.4)	299 (84.5)	
80–89	42 (23.7)	28 (7.9)	
≥90	37 (20.9)	27 (7.6)	
**Height categories (cm)**			**<0.001**
<160	68 (38.4)	201 (56.8)	
≥160	109 (61.6)	153 (43.2)	
**Maternal education level**			0.299
Primary	63 (35.6)	130 (36.7)	
Secondary	89 (50.3)	183 (51.7)	
Tertiary	25 (14.1)	41 (11.6)	
**Residence type**			0.147
Urban	152 (85.9)	319 (90.1)	
Rural	25 (14.1)	35 (9.9)	
**Marital status**			**0.011**
Single	11 (6.2)	48 (13.6)	
Married/ partnered	166 (93.8)	306 (86.4)	
**Religion**			0.681
Catholic	53 (29.9)	104 (29.4)	
Pentecostal/ Born Again	36 (20.3)	77 (21.7)	
Muslim	43 (24.3)	91 (25.7)	
Anglican	39 (22.0)	63 (17.8)	
Others (SDA, orthodox)	6 (3.4)	19 (5.4)	
**Working status**			0.028
Employed/ salaried	31 (17.5)	63 (17.8)	
Business	92 (52.0)	144 (40.7)	
Housewife/ Not working	54 (30.5)	147 (41.53)	

The following characteristics were significantly higher among the cases than in the controls; gestation age (39.5 ± 1.1 vs 39.0 ± 1.3, P value <0.001), ever delivered a baby greater than 4 kg (30.5% vs 12.4%, P value <0.001) and use of herbal medicine during pregnancy (33.9% versus 21.7%, P value = 0.003) (Table 2).

**Table 2:** Pregnancy and birth related characteristics.

	Cases n=177 (%)	Controls n=354 (%)	P value
Gestation age in weeks (mean ± SD), n = 478	39.5 ± 1.1	39.0 ± 1.3	**<0.001**
**Gestation age categories (weeks) n = 478**			**0.004**
37 - 39	83 (52,9)	213 (66.4)	
40 - 42	74 (47.1)	108 (33.6)	
**Parity (carried above 28 weeks)**			**0.002**
1	38 (21.5)	123 (34.8)	
2–4	107 (60.4)	192 (54.2)	
≥5	32 (18.1)	39 (11.0)	
**Ever delivered a baby >4 kg**			**<0.001**
No	123 (69.5)	310 (87.6)	
Yes	54 (30.5)	44 (12.4)	
**Number of deliveries >4 kg (n=98)**			0.996
1	36 (66.7)	30 (68.2)	
2	13 (24.1)	10 (22.7)	
3	4 (7.4)	3 (6.8)	
4	1 (1.9)	1 (2.3)	
**Ever had a still birth**			0.557
No	171 (96.6)	345 (97.5)	
Yes	6 (3.4)	9 (2.5)	
**Number of still births (n=15)**			0.143
1	4 (66.7)	8 (88.9)	
2	2 (33.3)	0 (0.0)	
3	0 (0.0)	1 (11.1)	
**Early neonatal death**			0.281
No	176 (99.4)	348 (98.3)	
Yes	1 (0.6)	6 (1.7)	
**Use of herbal medicine during pregnancy**			**0.003**
No	117 (66.1)	277 (78.3)	
Yes	60 (33.9)	77 (21.7)	

The mean Apgar scores at 5 minutes was significantly lower for cases as compared to controls (8.4 ± 2.6 vs 9.0 ± 2.0, P value = 0.009). In addition, the proportion of male babies born to the cases was significantly higher than that for the controls (63.8% vs 55.1%, P value = 0.049) (Table 3).

**Table 3:** Delivery and fetal characteristics.

	Cases n=177 (%)	Controls n=354 (%)	P value
Neonatal Apgar at 1 minute (mean ± SD)	7.2 ± 2.4	7.7 ± 1.9	**0.004**
Neonatal Apgar at 5 minutes (mean ± SD)	^8.4 ± 2.6^	^9.0 ± 2.0^	**0.009**
**Mode of delivery**			0.951
Vaginal	80 (45.2)	159 (44.9)	
Caesarean	97 (54.8)	195 (55.1)	
**Labour complications**			0.532
None	131 (74.0)	264 (74.6)	
Obstructed labor	24 (13.6)	39 (11.0)	
Ruptured uterus	7 (4.0)	10 (2.8)	
Others	15 (8.5)	41 (11.6)	
**Labour augmentation with oxytocin**			0.682
No	122 (70.9)	252 (72.6)	
Yes	50 (29.1)	95 (27.4)	
**Current delivery outcomes**			0.094
Live birth	164 (92.7)	340 (96.1)	
Still birth	13 (7.3)	14 (3.9)	
**Sex of the baby**			**0.049**
Male	113 (63.8)	195 (55.1)	
Female	63 (35.6)	159 (44.9)	
Sexual development disorder	1 (0.6)	0 (0.0)	

### Risk factors for fetal macrosomia

On multivariate analysis, the following factors were found to be risk factors for fetal macrosomia; maternal age ≥ 40 years, maternal weight ≥ 80 kg, maternal height ≥160 cm, being married, gestation age ≥ 40 weeks, history of having a prior delivery of a baby greater than 4 kg, use of herbal medicine during pregnancy, and having a male baby (Table 4).

**Table 4:** Multivariate analysis of maternal risk factors for fetal macrosomia.

	Cases n (%)	Controls n (%)	Adjusted OR (95%CI)	P value
**Number of participants**	177 (33.3)	354 (66.7)		
**Maternal age, year categories**				**0.020**
<30	109 (61.6)	264 (74.6)	Ref	
30–39	59 (33.3)	88 (24.9)	0.8 (0.49 - 1.38)	
≥40	9 (5.1)	2 (0.5)	7.4 (1.37 - 39.44)	
**Weight, kg categories**				**<0.001**
<80	98 (55.4)	299 (84.5)	Ref	
80–89	42 (23.7)	28 (7.9)	4.0 (2.15 - 7.40)	
≥90	37 (20.9)	27 (7.6)	3.9 (1.99 - 7.49)	
**Height, cm categories**				**0.040**
<160	68 (38.4)	201 (56.8)	Ref	
≥160	109 (61.6)	153 (43.2)	1.6 (1.02 - 2.51)	
**Marital status**				**0.038**
Single	11 (6.2)	48 (13.6)	Ref	
Married/ partnered	166 (93.8)	306 (86.4)	2.5 (1.05 - 5.95)	
**Gestation age, weeks categories**				**0.009**
37–39	83 (52.9)	213 (66.4)	Ref	
40–42	74 (47.1)	108 (33.6)	1.8 (1.16 - 2.82)	
**Ever delivered a baby >4 kg**				**0.006**
No	123 (69.5)	310 (87.6)	Ref	
Yes	54 (30.5)	44 (12.4)	2.2 (1.26 - 3.81)	
**Use of herbal medicine during pregnancy**				**0.001**
No	117 (66.1)	277 (78.3)	Ref	
Yes	60 (33.9)	77 (21.7)	2.3 (1.41 - 3.82)	
**Sex of the baby**				**0.011**
Female	63 (35.6)	159 (44.9)	Ref	
Male	113 (63.8)	195 (55.1)	1.78 (1.14 - 2.77)	

## Discussion

This was an unmatched hospital-based case control study to identify the risk factors for fetal macrosomia among women delivering at a National Referral Hospital in Kampala, Uganda. Risk factors for fetal macrosomia were- maternal age greater or equal to 40 years, maternal weight of 80 kg and above, height greater than 160 cm, being married, gestational age greater or equal to 40 weeks, history of previous macrosomic baby, use of herbal medicines during pregnancy and a male fetus.

The study found out that maternal age of 40 years and above had increased odds of fetal macrosomia. Mothers who were aged 40 years and above were 7.4 times more likely to deliver a macrosomic baby compared to those below 40 years These findings are consistent with a study done in Nigeria where the mean age of mothers that had macrosomic babies was reported to be significantly higher than that of mothers with normal birth weight babies [15]. This could be related to the frequent development of medical conditions like gestational diabetes mellitus in older women. This was however in disagreement with findings of a study done in South Africa which found that advanced maternal age women was associated with delivery of low birth weight babies [16]. This could also be explained by the increased frequency of conditions such as hypertension and gestational diabetes in pregnancy that can lead to fetal growth restriction [17]. There is literature pointing to the fact that placental development is abnormal in women of advanced maternal age due to an imbalance in angiogenic growth factors responsible for vascular development in the placenta which may cause fetal growth restriction [18].

Compared to women with maternal weight of 80 kg or less, women with a maternal weight of 80–89 kg had 4 times higher odds of delivering a macrosomic newborn (aOR = 4.0, 95%CI 2.15–7.40, P value <0.001). This could be attributed to increased metabolic fuels – hyper glycaemia, hyperlipidemia, increased amino acids in obese mothers that is broken down into glucose which is later transferred to the fetus across the placenta [19]. The findings from this study are similar to findings from studies done in Tanzania and Southeast Nigeria which reported an increased risk of macrosomia with increasing maternal weight [20,21].

Maternal height greater or equal to 160 cm was an independent risk factor for fetal macrosomia. Mothers whose height was greater or equal to 160 cm were 60 percent more likely to deliver macrosomic babies compared to mothers whose height was less than 160 cm These findings are similar to studies done in Nigeria and Saudi Arabia where it was reported that maternal height greater than 160 cm was significantly associated with macrosomia compared to controls [15,22–24]. This may be due to better nutrition since taller women are more likely to be better nourished than their shorter counterparts [25]. Additionally genetic factors may play a role as taller women are more likely to be bigger and therefore deliver bigger babies [26]. The odds of having a macrosomic infant were 2.5 times among the married women than in single women These findings are similar to results of studies done in Ethiopia and other low-and middle-income countries [6,27]. This may be attributed to better social and economic support to aid in nutrition and psychological wellbeing of the mother and generally good quality of life [28].

In this study, mothers whose gestational age was greater or equal to 40 weeks were 1.8 times more likely to deliver a macrosomic baby compared to those less than 40 weeks of gestation This is similar to findings by Koyanagi et al in his study entitled “macrosomia in 23 developing countries: an analysis of a multi-country, facility-based, cross-sectional survey” [5]. Adugna also found similar trends in his study done in Gondar, Ethiopia where mothers having a gestational age greater or equal to 40 weeks were 4.1 times more likely to be deliver macrosomic babies than mothers with less than 40 weeks [2]. This may be a result of a healthy, safe and satisfying pregnancy environment with consequent continued supply of nutrients and oxygen-rich blood to the developing fetus beyond 40 weeks. Fetal growth is also more rapid during the late stages of pregnancy as the fetal organs develop further and the fetus accumulates nutrients and fat stores which is a factor of time[29].

A previous history of delivering a macrosomic infant was found to be associated with 2.2 times odds of having another macrosomic infant compared to mothers with no prior history. This finding has been consistent across several studies [2,30,31]. This may be due to an inherited genetic tendency of a mother to deliver macrosomic babies. Use of herbal medicines during pregnancy was also found to be associated with macrosomia with a 2.3 times risk compared to mothers who never used herbal medicines herbs. This is in contrast to findings from other studies that did not report any association [32,33]. There seems to be no biologically plausible explanation except that these herbs may contain insulin inducing agents that may interfere with glucose metabolism hence leading to fetal macrosomia.

Delivery of a male infant was associated with macrosomia. Male babies were nearly 80 percent more likely to be macrosomic compared to their female counterparts. These findings are consistent with other studies done in Africa which found that male gender was significantly associated with increased risk of macrosomia [34,35]. Daily fetal growth appears to be higher in male fetuses than in females [36]. More weight gained by male babies in utero is thought to be a result of androgen action [37].

## Conclusions and recommendations

The risk factors for fetal macrosomia among women delivering at Kawempe National Referral Hospital in central Uganda were maternal age greater or equal to 40 years, maternal weight of 80 kg and above, height greater than 160 cm, gestational age greater or equal to 40 weeks, history of previous macrosomic baby, being married, use of herbal medicines during pregnancy and male fetus. It therefore becomes imperative to identify pregnant women at risk of fetal macrosomia during ANC, admission, labour, using simple processes such measuring maternal weight and height so that effective education, precautions and interventions are implemented. A well-designed protocol to guide in the management of mothers at risk of macrosomia would go a long way in reducing the adverse maternal and fetal outcomes such as shoulder dystocia, perineal trauma, fetal distress and many others that are known to be associated with fetal macrosomia.

## Study strengths and limitations

This study adds to a growing list of studies assessing risk factors for fetal macrosomia in Africa where most studies have been done outside East Africa. It therefore reinforces the body of knowledge on the subject of fetal macrosomia that is increasingly becoming more prevalent. Being a a case-control study it was possible to evaluate multiple risk factors associated with macrosomia.

One limitation of this study was that pregnancy weight gain could not be established since most mothers did not know their pre-pregnancy weight.
